# Advances in cancer nanomedicine

**DOI:** 10.7497/j.issn.2095-3941.2015.0049

**Published:** 2015-09

**Authors:** Lin-Tao Cai, Zong-Hai Sheng

**Affiliations:** Center for Nanomedicine and Nanobiotechnology, Institute of Biomedicine and Biotechnology, Shenzhen Institutes of Advanced Technology, Chinese Academy of Sciences, Shenzhen 518055, China

Cancer has become the leading cause of death. The progress in diagnosis and treatment is still limited. Over the past three decades, emergence and rapid development of nanotechnology have brought new hopes for cancer therapy.

A repertoire of nanomaterials with controllable size-, shape-, and composition- dependent physiochemical properties has been studied for drug delivery and biomarker detection. Microfluidic devices have been designed for circulating tumor cell isolation and sensitive diagnosis of biomarker panels. To date, more than 247 nanomaterials-based products have been approved by the Food and Drug Administration (FDA) in USA for clinical application^1^. With the constant emergency of novel nanomaterials, one of the biggest problems is how to translate nanomaterials from the laboratory to the clinic and the market.

To meet the challenges of nanotechnology in clinical transformation, several successful examples would provide some references for generations of biomedical nanomaterials. For example, gold nanoparticles-based spherical nucleic acids (SNAs) developed by Mirkin group^2^ at Northwestern University were utilized for intracellular mRNA detection, which led to the commercialization of NanoFlare technology under the trade name SmartFlares (Merck Millipore in partnership with AuraSense, LLC, Skokie, Illinois). Kataoka group^3^ at University of Tokyo prepared five different polymeric micelle drugs which have already been explored in clinical trials in Asia and the United States. Among them, paclitaxel micelle drug is in the final stage of phase III clinical trial in Japan for the treatment of recurrent breast cancer, and it is expected to proceed into the application for approval within a year. Valle *et al*.^4^ used Pluronic block copolymer micelle drug for the treatment of cancer (SP1049c) in phase II trials. Paithankar *et al*.^5^ at the University of California, Santa Barbara, used gold-coated silica nanoparticles for photothermal treatment of acne using low-frequency ultrasound as an enhanced delivery tool. The exciting achievements from clinical transformation of nanomaterials provided vast commercial opportunities. To accelerate the translation process, the following points should be addressed^6^: (I) a thorough understanding of the nanostructures and interaction between rational design of nanomaterials and physiological microenvironment; (II) molecular probes and engineered nanoprobes for preclinical and activatable prognosis treatment; (III) stimuli-responsive smart drug delivery systems for targeted therapy, especially clinical EPR effects; (IV) imaging guided therapy and surgery with theranostic nanoparticles; (V) safety considering, especially nanotoxicity *in vivo*; (VI) multi-disciplinary integration and multi-disciplinary team (MDT) cooperation for translational nanomedicine.

Cancer stem cells (CSCs) play a critical role in the tumor occurrence, deterioration, metastasis, and recurrence^7^. Specific targeting and removal of CSCs may be essential for the effective treatment and prevention of cancer. Nanomaterials as drug delivery system or imaging probe have been successfully applied for highly sensitive imaging and efficient therapy of CSCs^8^. Moreover, tumor microenvironment also allows for growth and metastasis of tumor cells, and aids in the resistance of cancer cells to current chemotherapy and radiotherapy^9^. Currently developed nanoprobe could selective bind with cancer cells and exhibit a high imaging sensitivity^10^. Therefore, nanotechnology is prone to design and synthesis of nanomedicine or nanoprobe for treatment of CSCs and tumor microenvironment.

By and large, with the quick development of nanotechnology, we need to advocate for a large scale multidisciplinary effort aiming at clinical transformation. We encourage more scientists to join in this exciting and prospective field.
